# Hospital Appointments

**Published:** 1859-07

**Authors:** 


HOSPITAL APPOINTMENTS.—The vacancy occasioned in the Pennsylvania Hospital by the resignation of Professor Wood, has been filled by the appointment of Dr. Francis G. Smith, a step which has given universal satisfaction.

The governors of the Bellevue Hospital, of New York, have added to the medical and surgical staff of this charity, Drs. A. B. Mott, W. H. Church, J. W. S. Gouley, and C. H. Meir, as Surgeons; and Drs. A. C. Loomis, and T. W. Greene, as Physicians.

Dr. J. B. Biddle has resigned the post of Attending Physician to the Episcopal Hospital of this city, and is succeeded by Dr. Henry Hartshorne.

